# An Examination of Motivation to Change and Neural Alcohol Cue Reactivity Following a Brief Intervention

**DOI:** 10.3389/fpsyt.2019.00408

**Published:** 2019-06-11

**Authors:** Erica N. Grodin, Aaron C. Lim, James MacKillop, Mitchell P. Karno, Lara A. Ray

**Affiliations:** ^1^Department of Psychology, University of California, Los Angeles, Los Angeles, CA, United States; ^2^Department of Psychiatry and Biobehavioral Sciences, UCLA School of Medicine, University of California, Los Angeles, Los Angeles, CA, United States; ^3^Department of Psychiatry and Behavioral Neurosciences, McMaster University, Hamilton, ON, Canada

**Keywords:** brief intervention, mechanisms of behavior change, motivation to change, alcohol, functional magnetic resonance imaging

## Abstract

**Background:** Brief interventions represent a promising psychological intervention targeting individuals with heavy alcohol use. Motivation to change represents an individual’s openness to engage in a behavior change strategy and is thought to be a crucial component of brief interventions. Neuroimaging techniques provide a translational tool to investigate the neurobiological mechanisms underlying potential mediators of treatment response, including motivation to change. Therefore, this study aimed to examine the effect of a brief intervention on motivation to change drinking behavior and neural alcohol taste cue reactivity.

**Methods:** Non–treatment-seeking heavy drinkers were randomized to receive a brief drinking intervention (n = 22) or an attention-matched control (n = 24). Three indices of motivation to change were assessed at baseline and after the intervention or control session: importance, confidence, and readiness. Immediately following the intervention or control session, participants also underwent an functional magnetic resonance imaging (fMRI) during which they completed an alcohol taste cues paradigm.

**Results:** There was a significant effect of the brief intervention on increasing ratings of importance of changing drinking behavior, but not on ratings of confidence or readiness to change. Ratings of importance after the intervention or control session were associated with neural alcohol taste cue reactivity, but notably, this effect was only significant for participants who received the intervention. Individuals in the intervention condition showed a positive association between ratings of importance and activation in the precuneus, posterior cingulate, and insula.

**Conclusions:** The brief drinking intervention was successful at improving one dimension of motivation to change among non–treatment-seeking heavy drinkers. The brief intervention moderated the relationship between ratings of importance and brain activation in circuitry associated with interoceptive awareness and self-reflection. Together, findings represent an initial step toward understanding the neurobiological mechanisms through which a brief intervention may improve motivation to change.

## Introduction

An increasing number of individuals engage in heavy alcohol use, putting themselves at risk of myriad health, psychological, and social consequences (1). Brief interventions represent a promising psychological intervention targeting individuals with heavy alcohol use who have not yet progressed to moderate or severe alcohol use disorder (AUD). Brief interventions are short (5 to 60 min), traditionally one to five sessions, interventions designed to increase motivation for behavioral change and encourage self-monitoring of high-risk situations for heavy drinking (2). Although specific therapeutic techniques vary, many of these interventions seek to increase motivation by providing individuals normative feedback about individualized risk of developing AUD, inquiring about the desire to change their drinking, and working collaboratively to explore and develop behavior change options (3). Meta-analyses have identified small yet robust effects of brief interventions on alcohol consumption that can be flexibly administered in multiple settings, including hospital emergency departments, primary care, and *via* digital/tele-therapy (2–4). Brief interventions have also been shown to sustain drinking reductions at 12-month follow-up (4).

Motivation for change is conceptualized as a multi-dimensional, dynamic construct representing one’s openness to engage in a behavior change strategy (5), and is thought to be a crucial component of brief interventions (6, 7). High levels of motivation for change have been considered a prerequisite for successful treatment response. For instance, among individuals with comorbid substance use disorders and serious mental illness, high motivation was associated with reporting greater cons and fewer benefits to using substances, and taking steps to reduce substance use (8). Motivation for change was also associated with higher client reports of therapeutic alliance with therapists among treatment-seeking problem drinkers (9). Among homeless individuals placed in a housing intervention program, motivation for change was a stronger predictor of alcohol outcomes than treatment attendance (10). Many brief interventions for AUD have, therefore, focused on enhancing motivation for change given its importance in treatment engagement and outcomes.

To advance the literature on behavior change applied to alcohol use, current scientific efforts have focused on elucidating the specific mechanisms of behavior change, including underlying neural-level substrates that subserve changes in alcohol use. To that end, neuroimaging techniques provide a translational tool to investigate the neurobiological mechanisms underlying potential moderators of treatment response. Several studies to date have used neuroimaging to probe the underlying neurobiological mechanisms of psychosocial interventions (11–14). Three studies have examined the mechanisms of motivational interviewing interventions in alcohol-using populations (11, 14, 15). These studies investigated the importance of client and therapist speech as components of motivational interview interventions. The first study found that client change talk was effective in attenuating neural reward response to alcohol cues (11). The second study found that the origin of client change language is crucial for motivational interventions; self-generated change talk and counter-change talk were associated with increased activation in brain regions associated with introspection and self-awareness, when contrasted with experimenter selected language (14). The third study found that therapist statements designed to encourage complex reflections were associated with neural response in brain regions associated with reward and self-reflection, when contrasted with closed questions from therapists (15). Together, these studies provide evidence that neuroimaging can be successfully used to investigate the neurobiological mechanisms of brief interventions for alcohol use.

Although motivation for behavioral change has been identified as a critical component of behavioral interventions, no translational studies have yet explored how the relationship between psychological interventions and motivational change are represented neurobiologically. Identifying a neurobiological substrate of a behavior change target, in this case motivation for change, is critical for understanding the mechanisms of behavior change (16, 17). There are several brain regions that may be involved in these processes, particularly those that are associated with incentive salience and introspection. Brain regions implicated in incentive salience processing in addictive disorders include the ventral striatum (nucleus accumbens), dorsal striatum (caudate and putamen), and the orbitofrontal cortex (18, 19). Brain regions involved in self-reflection and introspection include the posterior cingulate cortex, precuneus, and insula (12, 14).

We recently conducted a study designed to examine the effectiveness of a brief intervention on improving drinking outcomes and modulating neural alcohol cue reactivity (20). This study randomly assigned non–treatment-seeking heavy drinkers to receive a single-session brief intervention or to an attention-matched control condition. The brief intervention was designed to help participants understand their individual level of drinking risk and help initiate changes in their alcohol use. Participants completed an alcohol taste cue reactivity paradigm during a functional magnetic resonance imaging (fMRI) scan immediately following the intervention. Participants completed a follow-up visit one month after the intervention to report on their drinking behavior. There was no significant effect of the brief intervention on drinking outcomes at follow-up or on modulating neural alcohol taste cue reactivity.

A better understanding of the neurobiological mechanisms of how brief interventions work through motivational change may help improve treatments for alcohol using populations. Therefore, this secondary analysis (20) aimed to examine the effect of a brief intervention on motivation to change drinking behavior and neural alcohol taste cue reactivity. To do so, we first tested whether the brief intervention had an effect on proximal outcomes of motivation to change (i.e., readiness rulers). We hypothesized that participants in the brief intervention condition would exhibit greater motivation to change compared to the control group. We also examined the association between motivational readiness and alcohol taste cue reactivity and assessed if the brief intervention moderated this association. We hypothesized motivation to change would be positively related to neural alcohol cue reactivity in circuitry associated with introspection and self-reflection and negatively related to neural alcohol cue reactivity in regions implicated in reward and incentive salience. We further hypothesized that these relationships would be stronger in the intervention condition compared to the control condition.

## Materials and Methods

### Participants and Screening Procedures

The study protocol and all procedures were approved by the Institutional Review Board of the University of California, Los Angeles. Detailed methodology of the general screening and experimental procedures has been published elsewhere (20) and are summarized here. Interested participants completed an initial telephone interview and eligible participants were invited to participate in an in-person screening visit. Upon arrival, all participants read and signed an informed consent form in accordance with the Declaration of Helsinki. During the in-person screening visit, participants completed a psychiatric diagnostic interview and a battery of individual difference measures, including demographics and alcohol and drug use assessments. All participants were required to have a breath alcohol concentration of 0.000 g/dl and to test negative on a urine drug test (except for marijuana, which was allowed to be positive).

Participants were non–treatment-seeking heavy drinkers, indicated by consuming five or more drinks per occasion for men or four or more drinks per occasion for women at least four times in the month preceding study enrollment, and who scored at least an 8 on the Alcohol Use Disorder Identification Test (AUDIT) (21). A total of 120 participants were screened in the laboratory for eligibility; 38 did not meet inclusion criteria, and 12 elected not to participate, leaving 60 participants who were enrolled and randomized. Of the 60 participants randomized, 46 participants completed the entire study. Participants who completed all study visits were compensated US $160.

### Study Design

Participants were assessed at three time-points: at baseline, at randomization, and 1-month follow-up. During the randomization visit, participants were randomly assigned to receive a one-session brief drinking intervention or to an attention-matched control condition. Immediately following the intervention or control session, participants completed an fMRI scan to assess brain activity during exposure to alcohol and water taste cues. Participants returned for a follow-up visit approximately 4 weeks after the intervention or control session to assess alcohol use.

The brief intervention consisted of a 30- to 45-min individual face-to-face session based on the principles of motivational interviewing (22, 23) and adhered to the FRAMES model, which includes personal feedback (F), emphasizing personal responsibility (R), providing brief advice (A), offering a menu (M) of change options, conveying empathy (E), and encouraging self-efficacy (S). The aim of the intervention was to help participants understand their level of risk and to help initiate changes in their alcohol use. Participants randomized to the attention-matched control condition viewed a 30-min video about astronomy. In the control condition, there was no mention of alcohol or drug use beyond completion of research assessments.

### Individual Difference Measures

The following individual questionnaires and interviews were administered during the study: 1) the 30-day timeline follow-back (TLFB) was administered in interview format to capture daily alcohol use over the 30 days prior to the visit (24), 2) the self-report AUDIT was administered to assess for drinking severity (21), and 3) the Penn Alcohol Craving Scale (PACS) was administered to assess alcohol craving (25). Participants also completed the Fagerstrom Test for Nicotine Dependence (26). The Structured Clinical Interview for DSM-5 (SCID) (27) was administered by a clinician to assess for lifetime and current AUD. Lastly, participants completed a demographics questionnaire reporting, among other variables, age, sex, and level of education.

### Motivation to Change Assessment

At each visit, participants also completed three decision rulers designed to measure their motivation to change their drinking behavior [based on Refs. (5, 28)]. Participants were asked to rate on a scale from 1 to 10: “As of now how important is it for you to make a change in your drinking?” (importance ruler), “If you decided to make a change in your drinking how confident are you that you could do it?” (confidence ruler), and “As of now how ready are you to make a change in your drinking?” (readiness ruler).

### Neuroimaging Procedures

At the start of the scanning visit, participants were required to have a BrAC of 0.00 g/dl and a urine toxicology screen negative for all drugs (excluding tetrahydrocannabinol). Additionally, female participants were required to have a negative pregnancy test.

Neuroimaging data were acquired on a 3.0T Siemens Prisma scanner at the UCLA Staglin Center for Cognitive Neuroscience. Detailed neuroimaging parameters can be found in Grodin et al. (20). Briefly, the protocol consisted of a high-resolution, matched-bandwidth (MBW) scan and a structural magnetization-prepared rapid-acquisition gradient echo (MPRAGE) scan. This was followed by two runs of a modified version of the Alcohol Cues Task, which involves the delivery of oral alcohol or control (water) tastes to elicit physiological reward responses (29, 30). During the task, participants were presented with a visual cue indicating the trial type (Alcohol Taste or Water Taste), which was followed by a fixation cue and the delivery of the alcohol or water taste (1 ml).

Preprocessing of the neuroimaging data followed conventional procedures implemented in FMRIB’s Software Library (FSL 5.0) (www.fmrib.ox.ac.uk/fsl). This included motion correction [Motion Correction Linear Image Registration Tool (McFLIRT, Version 5.0)], high-pass temporal filtering (100-s cutoff) using FSL’s FMRI Expert Analysis Tool (FEAT, Version 6.00), and smoothing with a 5-mm full-width at half-maximum Gaussian kernel. FSL’s Brain Extract Tool (BET) was used to remove skull and non-brain tissue from both the structural and functional scans. Data were denoised using ICA-AROMA (31) to reduce motion artifacts associated with swallowing.

### Data Analysis

General linear models with OLS regression were used to test the main effect of study condition on each of the three motivation-for-change decision rulers (importance, confidence, and readiness). Analyses were adjusted for baseline AUDIT score, age, sex, smoking status, and the baseline ratings from the corresponding decision ruler.

The analysis of the Alcohol Cues Task was conducted using FSL’s FEAT as described in Ref. (20). Briefly, alcohol and water taste cues were convolved with a double-gamma hemodynamic response function (HRF). Six motion regressors representing translational and rotational head movement were included as regressors of no interest. Data for each subject were registered to the MBW, followed by the MPRAGE using affine linear transformations, and then were normalized to the Montreal Neurological Institute (MNI avg152) template. Registration was refined using FSL’s non-linear registration tool. The primary contrast of interest, the Alcohol Taste Cue > Control Taste Cue contrast, was defined in the first-level models. The second-level model combined the contrast images across the two task runs, within subjects. The third-level model combined the contrast images between subjects. To evaluate if the intervention moderated the association between motivational readiness ratings and brain activation to alcohol taste cues, three interaction models were run with baseline-corrected ratings of importance, confidence, and readiness mean-centered across all subjects. Age, sex, cigarette smoking status, positive urine for tetrahydrocannabinol (THC), and AUD severity were entered as covariates. Z-statistic images were thresholded using a cluster threshold of Z > 2.3 and a (corrected) cluster significance threshold of *P* < 0.05 (32). Given the exploratory nature of this study and the dearth of studies on behavioral interventions, neural reactivity to alcohol cues, and mechanisms of motivation to change, we also implemented a more restrictive approach presented in the **Supplementary Materials**. Specifically, we conducted a separate set of analyses using the regions significantly activated in the Alcohol Taste Cue > Water Taste Cue contrast as a mask to investigate if the intervention moderated the association between motivation to change ratings and task-specific brain activation. As the neuroimaging literature has not reached a standard whereby such masks are systematically used to test treatment effects, we provide both approaches in this manuscript (33).

## Results

### Effect of Brief Intervention on Motivation to Change Ratings

The groups significantly differed on their post-session ratings of importance (*F*_1,40_ = 8.77, *p* = 0.005), after controlling for age, gender, smoking status, and baseline ratings of importance. Specifically, the intervention group had higher post-session ratings of importance than the control group (intervention group, 6.27 ± 0.39; control group, 4.67 ± 0.37; predicted values). However, there was no significant effect of group on ratings of confidence (*F*_1,40_ = 1.35, *p* = 0.25; intervention group: 7.13 ± 0.44; control group: 6.25 ± 0.42; predicted values) or readiness (*F*_1,40_ = 0.04; *p* = 0.85; intervention group, 4.73 ± 0.48; control group, 4.62 ± 0.43; predicted values) following the intervention or control sessions (see **Table 1**).

**Table 1:** Participant characteristics.

Characteristics	Intervention group (n = 22)	Control group (n = 24)	Statistic	p
Age	36.41 ± 13.56	32.29 ± 9.89	t = 1.18	0.24
Sex (M/F)	13/9	15/9	χ^2^ = 0.06	0.81
Cigarette smokers (n)	11	12	χ^2^ = 0.00	1
THC positive (n)	6	6	χ^2^ = 0.04	0.86
Education (years)	15.45 ± 2.13	15.04 ± 1.78	t = 0.72	0.48
AUDIT total score	17.68 ± 6.49	17.17 ± 7.61	t = 0.25	0.81
PACS score	19.32 ± 6.94	18.79 ± 7.15	t = 0.25	0.80
AUD severity (no diagnosis/mild/moderate/severe)	1/9/5/7	5/8/5/6	χ^2^ = 0.95	0.34
*Baseline Visit Motivation Ruler Ratings (T1)*				
Importance ruler	4.27 ± 2.53	5.25 ± 2.80	t = 1.21	0.23
Confidence ruler	5.68 ± 2.67	6.08 ± 2.43	t = 0.52	0.60
Readiness ruler	3.23 ± 1.88	3.88 ± 2.01	t = 1.10	0.28

### Relationship of Motivation to Change and Neural Alcohol Taste Cue Reactivity

#### Importance Ruler

**Figure 1:**
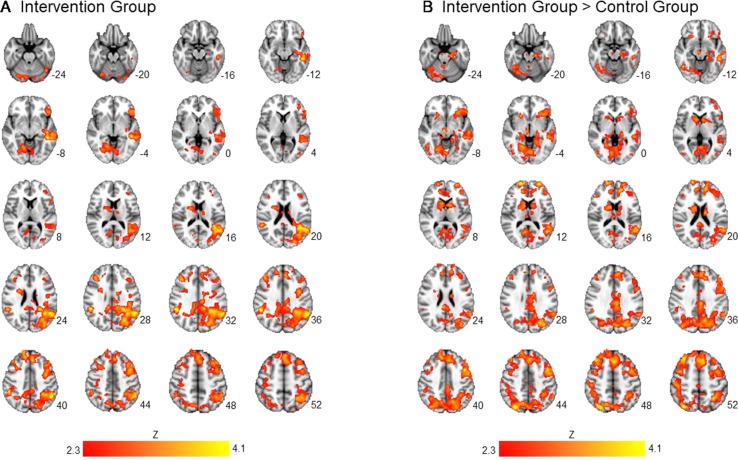
Association between importance ratings and brain activation to alcohol taste cues. The association between ratings of importance of behavioral change and brain activation to alcohol taste cues. **(A)** The intervention group showed a significant positive association between ratings of importance and brain activation in the precuneus, posterior cingulate, and caudate. **(B)** Between groups, the intervention group showed a significant association between importance ratings and brain activation in the posterior cingulate, insula, precuneus, caudate, and anterior cingulate. These associations were not present in the control group. See **Table 2** for a full list of significant regions. Z-statistic maps are whole-brain cluster corrected, Z > 2.3, p < 0.05. Coordinates are in Montreal Neurological Institute (MNI) space. Brain is displayed in radiological convention (L = R).

Averaging across intervention and control groups, there was no significant association between importance ratings and brain activation to alcohol taste cues. However, consistent with our hypothesis, there was a significant interaction between group and importance ratings on brain activation to alcohol vs. water taste. Specifically, there was a positive association between importance ratings and brain activation in frontal, limbic, and visual regions in the active intervention group (*p* < 0.05 corrected), whereas there was no significant association in the control group (see **Figure 1**, **Table 2**).

**Table 2:** association between importance ratings and brain activation to alcohol vs. water taste cues in intervention and control groups.

Brain region	Cluster voxels	Max. Z	x	y	z
Intervention group positive					
L Middle temporal gyrus	10,401	4.34	−46	−36	−10
L Angular gyrus		4.13	−52	−52	36
L Posterior cingulate gyrus		3.55	−14	−40	32
R Posterior cingulate gyrus		3.42	10	−40	28
R Precuneus		3.08	16	−70	50
L Middle frontal gyrus	4,187	3.90	−42	6	46
L Superior frontal gyrus		3.80	−4	24	48
L Cerebellar pyramis	2,374	4.49	−22	−80	−36
R Caudate	1,142	4.31	22	2	20
R Middle frontal gyrus		3.47	42	34	36
Control group positive					
N/A					
Intervention group negative					
N/A					
Control group negative					
N/A					
Intervention group > control group					
R Precuneus	11,068	4.69	32	−72	48
R/L posterior cingulate		3.64	−6	−24	34
L Precuneus		3.76	−14	−64	36
L Caudate		3.23	−10	8	10
R Lateral occipital cortex		3.11	26	−722	34
L middle frontal gyrus	7,647	4.11	−44	10	40
L frontal pole		3.83	−24	62	12
L superior frontal gyrus		3.57	−10	20	56
R/L anterior cingulate		3.46	16	42	10
L insula		3.17	−28	24	−4
R caudate	865	4.43	20	2	20
Control group > intervention group					
N/A					

For the analyses restricted to the mask representing significant clusters for Alcohol Taste Cue > Control Taste Cue, averaging across intervention, and control groups, there was no significant association between importance ratings and brain activation masked within the alcohol taste cue > water taste cue contrast. There was a significant interaction between group and importance ratings on brain activation to alcohol vs. water taste. Specifically, there was a positive association between importance ratings and brain activation in frontal regions, including the middle and superior frontal gyri and paracingulate, in the active intervention group (*p* < 0.05 corrected), whereas there was no significant association in the control group (see **Figure S1**, **Table S1**).

#### Confidence Ruler

There were no significant associations between ratings of confidence and brain activation to alcohol taste cues across or between groups. There was also no significant interaction between group and confidence ratings on neural alcohol taste cue reactivity.

For the masked analyses, there were no significant associations between ratings of confidence and masked brain activation to alcohol taste cues across or between groups. There was also no significant interaction between group and confidence ratings on masked neural alcohol taste cue reactivity.

#### Readiness Ruler

**Figure 2:**
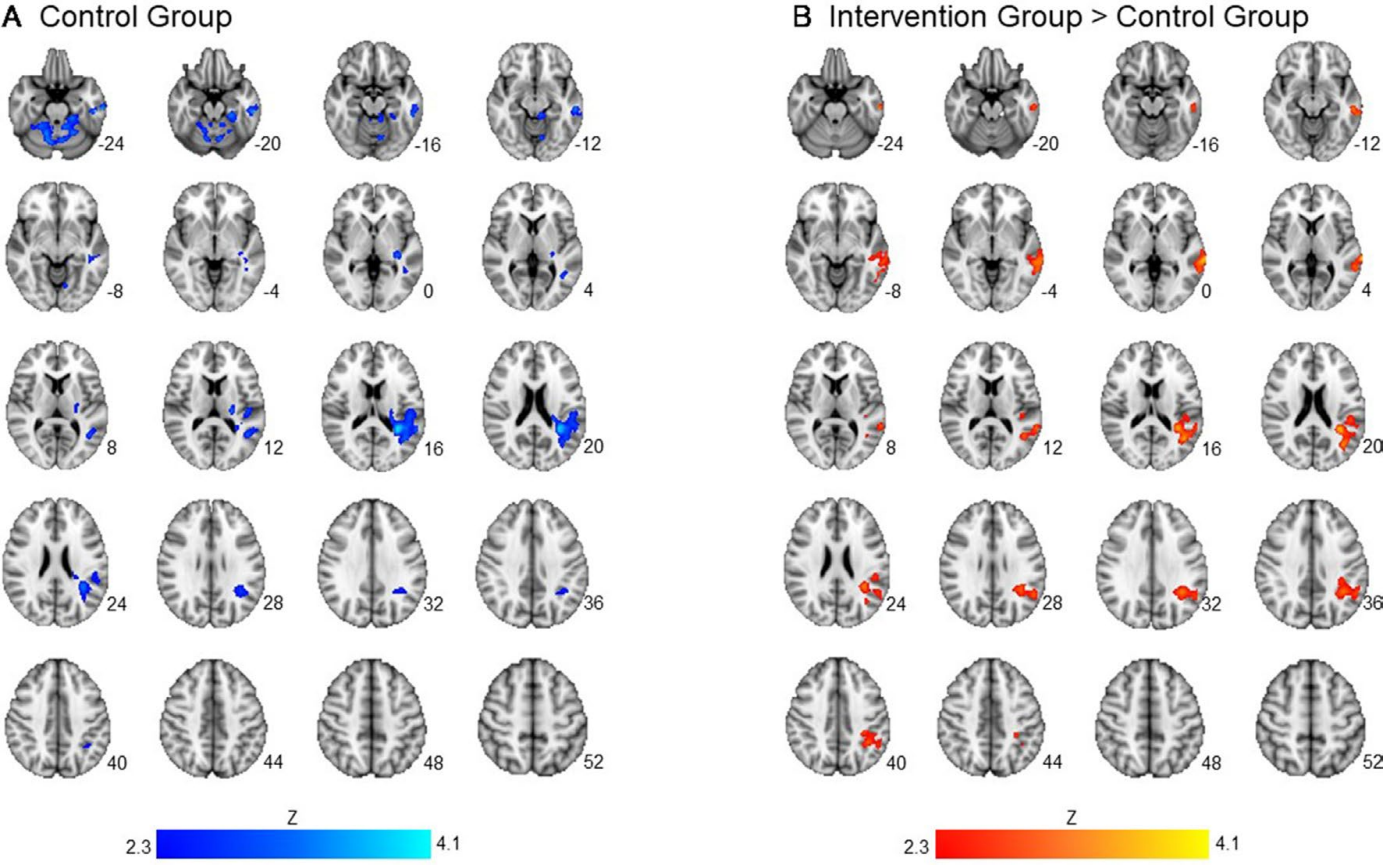
Association between readiness ratings and brain activation to alcohol taste cues. The association between ratings of readiness to change and brain activation to alcohol taste cues. **(A)** The control group showed a significant negative association between ratings of readiness and brain activation in the temporal lobe. **(B)** Between groups, the intervention group showed a significantly greater activation in the temporal lobe due to the negative relationship found in the control group. See **Table 3** for a full list of significant regions. Z-statistic maps are whole-brain cluster corrected, *Z* > 2.3, *p* < 0.05. Coordinates are in MNI space. Brain is displayed in radiological convention (L = R).

Across groups, there was no significant association between readiness ratings and brain activation to alcohol taste cues. There was a significant interaction between group and readiness ratings on neural activation to alcohol taste cues in the temporal lobe. Specifically, the control group showed a negative association between ratings of readiness to change and brain activation in the middle and superior temporal gyrus (*p* < 0.05 corrected). There was no significant association, positive or negative, between ratings of readiness to change and brain activation to alcohol cues in the intervention group (see **Figure 2**, **Table 3**).

**Table 3:** Association between readiness ratings and brain activation to alcohol vs. water taste cues in intervention and control groups.

Brain region	Cluster voxels	Max. Z	x	y	z
Intervention group positive					
N/A					
Control group positive					
N/A					
Intervention group negative					
N/A					
Control group negative					
R cerebellar tonsil	2,385	3.50	24	−66	−36
L superior temporal gyrus	2,232	3.96	−32	−44	18
L middle temporal gyrus		2.82	−60	−24	−18
Intervention group > control group					
L middle temporal gyrus	2,660	3.78	−66	−36	0
Control group > intervention group					
N/A					

For the masked analyses, there were no significant associations between ratings of readiness and masked brain activation to alcohol taste cues across or between groups. There was also no significant interaction between group and confidence ratings on masked neural alcohol taste cue reactivity.

## Discussion

This study examined the effect of a brief intervention on motivation to change, as indicated by ratings of importance, confidence, and readiness, in a sample of non–treatment-seeking heavy drinkers. This study also explored the relationship between indices of motivation to change and the neural substrates of alcohol taste cue-reactivity after a brief drinking intervention. We found that the brief intervention was successful in significantly increasing ratings of importance of changing behavior related to alcohol use. However, there was no effect of the intervention on ratings of confidence or readiness to change. Correspondingly, we found that the brief intervention moderated the association between ratings of importance of behavioral change and neural alcohol taste cue reactivity. Specifically, there was a significant positive association between ratings of importance and neural alcohol taste cue reactivity in regions associated with introspection and self-awareness in the intervention group, but not in the control group.

One goal of this study was to explicitly test if the brief intervention was effective at impacting motivation to change indices, which may serve as mechanism of behavior change (MOBC) (17). As we hypothesized, the brief drinking intervention increased ratings of importance of behavioral change. The intervention did not, however, impact ratings of confidence or readiness. Notably, as reported elsewhere, there was no significant main effect of the intervention on alcohol outcomes in the 4 weeks following the brief intervention (20). Therefore, it may not be surprising that the intervention was also not successful at increasing ratings of confidence or readiness to change. Importance, confidence, and readiness measure different elements of the change process, with each element being necessary, but not sufficient to induce a behavioral change (10, 22, 28). These results are similar to those of a motivational interview study among young adults admitted to an emergency room who reported risky drinking *via* the AUDIT or exhibited elevated blood alcohol content (34). In this study, a motivational interview, relative to personalized feedback alone, increased readiness to change ratings only at a trend level, and readiness to change did not mediate treatment effects on drinking outcomes. By contrast, adult emergency department heavy drinkers randomized to receive brief intervention relative to those receiving standard care reported higher readiness scores at 3 months post-treatment (35), and readiness mediated intervention effects only among those with high baseline motivation to change. Changes in readiness to change have also been shown to mediate brief intervention effects among underage heavy drinkers (36). Overall, these findings corroborate potential mechanisms of action of brief intervention, and may also explain the relatively small effect sizes reported in meta-analyses (2). Further, these results extend the literature by suggesting that neuroimaging tools, and cue reactivity in particular, were sensitive to changes in importance ratings, despite the fact that such changes did not lead to detectable treatment effects on alcohol use.

Notably, there is significant heterogeneity in measures utilized in the literature to capture readiness to change, with varying number of factors included in an instrument [e.g., Contemplation Ladder (37)], without widespread consensus on associations among measures. In light of these differences, studies utilizing the three ladders in this study suggest that baseline importance and confidence rather than readiness predict favorable drinking outcomes at 6 months post-brief intervention (38, 39). However, another study monitoring measures of readiness to change using these ladders found significant effects of confidence and readiness ratings on 12-month alcohol outcomes, with weaker effects of importance of change (40). Other brief intervention studies, however, have identified that baseline perception of alcohol-related problems is predictive of *greater* drinking 3 months later, whereas “Taking Action” ratings and having a personalized plan for change were significant predictors of reduced drinking 3 and 12 months later, respectively (41, 42). In light of this mixed literature, the findings for the present study may provide evidence for a modest effect of brief interventions on at least one dimension of readiness to change among non–treatment-seeking adult heavy drinkers. Additional research is needed to examine the clinical utility of the importance measure, as well as its overlap with other readiness to change assessments. Within this mixed literature, however, what remains more consistently corroborated is that alterations in importance ratings alone are insufficient to produce behavioral change. Within an MOBC context, the brief intervention within this study was successful at increasing the recognition of the importance of changing drinking behavior, when compared with the attention-matched control. Similarly, as the intervention was not successful in increasing ratings of confidence or readiness or in reducing drinking reported at follow-up, the brief behavioral intervention may need to be better modified to target these motivation to change ladders in efforts to induce reductions in drinking. Furthermore, there is evidence to suggest that during brief interventions, patients who set clear objectives for alcohol use reduction have better alcohol use outcomes over 12 months (43). These individuals also engaged in more change talk during the intervention and had higher ratings of importance and readiness to change (43). These results suggest that targeting patient goals for alcohol reduction may improve outcomes, potentially through motivation to change mechanisms.

The present findings did not support an overall association between motivation to change and neural alcohol taste cue reactivity; however, they did identify a moderating effect of the brief intervention on the relationship between motivation to change and neural alcohol taste cue reactivity. More specifically, we found that in the intervention group, but not in the control group, there was a significant positive association between ratings of importance of behavioral change and neural alcohol taste cue reactivity in regions implicated in introspection and self-reflection, e.g., precuneus, posterior cingulate, insula. Several studies have identified a role for the precuneus and the insula in self-related cognitive processes (44–46). Our findings are in line with other studies which have found increases in the recruitment of interoceptive and self-referential processing regions in response to motivational interventions (14, 47–50). Addictive disorders have been theorized to be associated with a deficit in insight and self-awareness (51) and metacognitive processing (52, 53). Therefore, the brief intervention’s emphasis on personalized level of risk and focus on change may have allowed individuals to increase their awareness of their drinking problems, thereby activating brain regions associated with interoceptive awareness when exposed to alcohol taste cues. In contrast, the control group, who did not receive personalized feedback, did not show an association between importance of behavioral change and activation in interoceptive circuitry.

This pattern of findings suggests a potentially important role of self-reflection in brief intervention and the neurobiology of alcohol cue reactivity. To wit, self-reflection during the intervention may have yielded higher problem awareness (i.e., importance for change). This self-reflection generalized to the scanning environment, wherein problem awareness prompted by the intervention was associated with greater introspection in response to alcohol cues. In contrast, participants in the control group did not engage in a self-reflective process about their drinking before the scanning session, and for them, the rating of importance was not associated with greater introspection in response to alcohol cues. These findings imply that it matters how people arrive at varying states of motivational readiness and that people who engage in self-reflection and also rate high on importance for change are the ones most likely to respond to subsequent alcohol cues with introspection. Future analyses should examine how these processes relate to alcohol use.

There was also a significant moderating effect of the brief intervention on the association between importance ratings and neural alcohol taste cue reactivity in regions implicated in incentive reward processing. The intervention group, when contrasted with the control group, showed a significant positive association between importance ratings and neural alcohol taste cue reactivity in the caudate, anterior cingulate, and insula, key regions of the incentive reward network (54). Intriguingly, the anterior cingulate is also implicated in monitoring conflict (55, 56). The activation of the anterior cingulate may represent the conflict between personal realizations of the importance of changing drinking behavior and the alcohol cue-elicited craving responses in incentive reward regions. Notably, the neuroimaging results using the mask-based approach did not fully conform with the pattern of findings from whole brain analyses discussed herein, and more broadly, did not address the study hypotheses given that the task contrast mask did not include brain regions subserving interoception.

Although the effects on the importance ratings were consistent with our prediction, this study also yielded a counterintuitive finding with regard to the association between neural activation to alcohol taste cues and the readiness to change ratings. Specifically, we found a significant interaction between group and post-session readiness ratings on neural activation to alcohol taste cues in the temporal lobe, such that the control group showed a negative association between ratings of readiness to change and brain activation in the middle and superior temporal gyrus. In the intervention group, however, there was no significant association, positive or negative, between ratings of readiness to change and brain activation to alcohol cues. In interpreting these findings, we considered two possibilities. The first is that this may be a spurious finding or type II error. The second possibility is that in fact these results reflect underlying effects such that in the control group, readiness to change was associated with decreased neural activation in the superior temporal gyrus during alcohol taste cues, compared to neural cues. We choose to refrain from reverse inference (57) in this case and note that additional studies and/or advanced data modeling may be required (58) to fully unpack this counterintuitive finding. Nonetheless, this result allows us to ponder on the very nature of this thematic issue, which is the degree to which clinical phenomenon will lend itself to cognitive neuroscience examination. Specifically, by breaking down clinical phenomena too finely we may lose its clinical significance, whereas having “large chunks” of clinical data explained by neuroimaging may lead to inconclusive or unreliable findings (59).

This study represents an initial step toward understanding the neurobiological mechanisms through which a brief intervention may improve motivation to change. Although this study has several strengths, it should be considered in light of its limitations. First, this study has a modest sample size; future studies should recruit larger sample sizes, particularly as the effect sizes of brief interventions are modest (60). Relatedly, this study recruited and enrolled non–treatment-seeking individuals from the community, and therefore, may not have shown the same changes in motivation to change following a psychosocial intervention as a treatment-seeking sample, which in turn may have reduced our power to identify associations between measures of readiness to change and neural alcohol cue reactivity. Additionally, the scanning portion of the study did not employ a pre-/post-treatment design, which may have been more sensitive to the effects of the intervention.

In conclusion, this study sought to identify the neurobiological mechanisms underlying changes in motivation induced by a brief intervention in non–treatment-seeking heavy drinkers. The current study found that a brief intervention increased ratings of importance of behavioral change, but was unsuccessful in impacting ratings of confidence or readiness to change compared to an attention-matched control. The brief intervention also moderated the association between neural alcohol taste cue reactivity and ratings of importance, such that in the intervention condition, there was a significant, positive relationship between ratings of importance and activation in regions associated with interoceptive awareness and self-reflection. This association may provide initial support for the role of interoceptive circuitry subserving increases in understanding of importance of behavioral change.

## Ethics Statement

The study protocol and all procedures were approved by the Institutional Review Board of the University of California, Los Angeles. Detailed methodology of the general screening and experimental procedures has been published elsewhere (20) and are summarized here. Interested participants completed an initial telephone interview and eligible participants were invited to participate in an in-person screening visit. Upon arrival, all participants read and signed an informed consent form in accordance with the Declaration of Helsinki.

## Author Contributions

MK, LR, and JM were responsible for the study concept and design. AL and LR conducted the study. EG analyzed the data. EG and AL wrote the manuscript. MK, LR, and JM provided critical feedback on the manuscript. All authors critically reviewed content and approved the final version for publication.

## Funding

This study was supported by NIH grant R21AA023669 (LR and MK) and training grant T32DA024635 (EG and AL).

## Conflict of Interest Statement

The authors declare that the research was conducted in the absence of any commercial or financial relationships that could be construed as a potential conflict of interest.
