# Supplementary material for: An Examination of Motivation to Change and Neural Alcohol Cue Reactivity Following a Brief Intervention
Source: Front Psychiatry. 2019 Jun 11;10:408. doi: 10.3389/fpsyt.2019.00408 (PMC6580427; doi:10.3389/fpsyt.2019.00408)
Supplement: Supplementary file 1 [file DataSheet_1.docx]

**Supplementary Material for: An Examination of Motivation to Change and Neural Alcohol Cue Reactivity Following a Brief Intervention**

**Results:**

**Relationship of Motivation to Change and Neural Alcohol Taste Cue Reactivity (Task Contrast Masked):**

*Importance Ruler*

Averaging across intervention and control groups there was no significant association between importance ratings and brain activation masked within the alcohol taste cue > water taste cue contrast. There was a significant interaction between group and importance ratings on brain activation to alcohol vs. water taste. Specifically, there was a positive association between importance ratings and brain activation in frontal regions, including the middle and superior frontal gyri and paracingulate, in the active intervention group (p < 0.05 corrected), while there was no significant association in the control group (see Figure S1, Table S1).

*Confidence Ruler*

There were no significant associations between ratings of confidence and masked brain activation to alcohol taste cues across or between groups. There was also no significant interaction between group and confidence ratings on masked neural alcohol taste cue reactivity.

*Readiness Ruler*

There were no significant associations between ratings of readiness and masked brain activation to alcohol taste cues across or between groups. There was also no significant interaction between group and confidence ratings on masked neural alcohol taste cue reactivity.

**Table S1. Association Between Importance Ratings and Masked Brain Activation to Alcohol vs. Water Taste Cues**

| Brain Region | Cluster Voxels | Max. Z | x | y | Z |
| --- | --- | --- | --- | --- | --- |
| Intervention Group Positive |  |  |  |  |  |
| L Superior Frontal Gyrus | 1,218 | 3.70 | -4 | 36 | 42 |
| L Middle Frontal Gyrus |  | 3.43 | -38 | 24 | 42 |
| L Paracingulate Gyrus |  | 3.12 | -8 | 36 | 34 |
|  |  |  |  |  |  |
| Control Group Positive |  |  |  |  |  |
| N/A |  |  |  |  |  |
|  |  |  |  |  |  |
| Intervention Group Negative |  |  |  |  |  |
| N/A |  |  |  |  |  |
|  |  |  |  |  |  |
| Control Group Negative |  |  |  |  |  |
| N/A |  |  |  |  |  |
|  |  |  |  |  |  |
| Intervention Group > Control Group | |  |  |  |  |
| L Superior Frontal Gyrus | 2,195 | 4.01 | -24 | 10 | 62 |
| L Paracingulate Gyrus |  | 3.57 | -4 | 34 | 40 |
| L Middle Frontal Gyrus |  | 3.15 | -42 | -2 | 62 |
|  |  |  |  |  |  |
| Control Group > Intervention Group | |  |  |  |  |
| N/A |  |  |  |  |  |

**
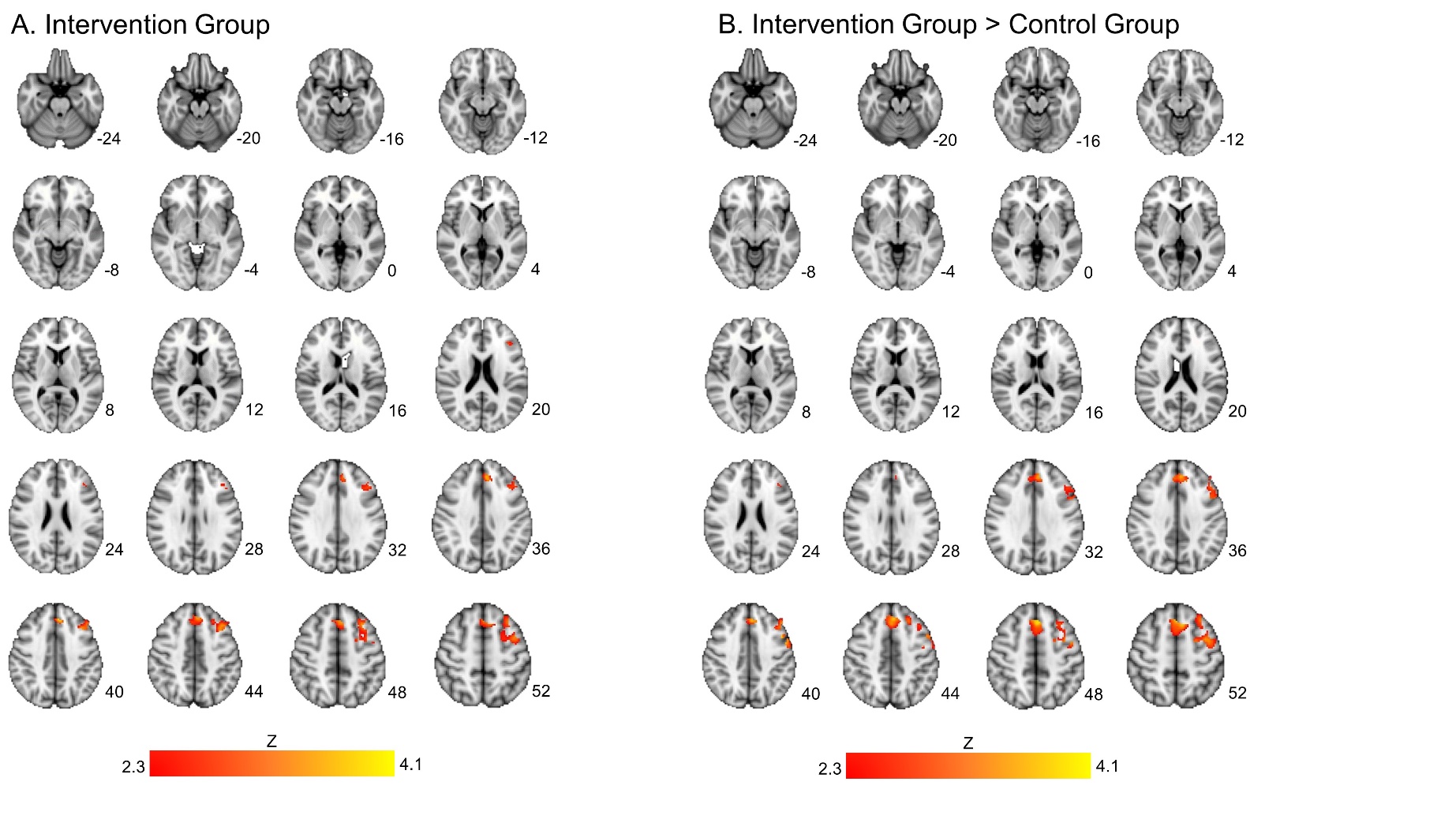
Figure S1. Association between Importance Ratings and Masked Brain Activation to Alcohol Taste Cues**

The association between ratings of importance of behavioral change and masked brain activation to alcohol taste cues. A. The intervention group showed a significant positive association between ratings of importance and brain activation in the middle and superior frontal gyri. B. Between groups, the intervention group showed a significant association between importance ratings and brain activation in the middle and superior frontal gyri. These associations were not present in the control group. See Table S1 for a list of significant regions. Z-statistic maps are whole-brain cluster corrected, Z > 2.3, p = 0.05. Coordinates are in MNI space. Brain is displayed in radiological convention (L=R).
